# Bilingual teachers’ translanguaging practices and ideologies in online classrooms in Saudi Arabia

**DOI:** 10.1016/j.heliyon.2022.e10537

**Published:** 2022-09-06

**Authors:** Muhammad Alasmari, Fawaz Qasem, Rashad Ahmed, Muhammad Alrayes

**Affiliations:** a Department of English, College of Sciences and Arts, University of Bisha, Al-Namas, Saudi Arabia; b Jacksonville State University, USA; c English Language and Literature Department, College of Arts, King Saud University, Riyadh, Saudi Arabia

**Keywords:** Bilingual teachers, Online classroom, Translanguaging practices, Translanguaging perspectives

## Abstract

Prior studies in translanguaging have investigated its role in education from different perspectives to understand its efficiency, practicality, and how it promotes or challenges educational and societal aims in different multilingual contexts across the world. However, little attention has been paid to translanguaging in universities with a monolingual environment. To cover this gap, the current study examines teachers' online translanguaging practices and ideologies in Saudi Arabia, where the community language is Arabic but English is commonly a medium of instruction in higher education. The study investigated (a) teachers' practices and perspectives toward translanguaging while communicating online with learners; and (b) how, when, and where teachers find translanguaging to be productive. The study adopts a mixed-methods approach to survey 260 bilingual instructors from universities in Saudi Arabia. In addition, 20 teachers’ video-recorded sessions are observed to assess the functions of translanguaging during online synchronous instruction. Five of these teachers are interviewed using stimulated-recall techniques. Results show that the teachers mostly hold positive views about translanguaging, considering it productive in helping students understand complex terms and in engaging in communication inside and outside the classroom. The data suggests that bilingual teachers of Arabic and English prefer the new bilingual approach of translanguaging and appear to depend less on the traditional monolingual approach to teaching in multilingual contexts.

## 1 Introduction

Educational institutions around the globe witnessed a rapid shift to online teaching during the COVID-19 pandemic. Saudi Arabia, for example, shifted all education online from 3 March 2020 to the end of that academic year [1]. According to the World Economic Forum [2], as a result of this dramatic shift, online learning might be here to stay its benefits such as an increase in retention of information [3]. Such a large-scale change requires an examination of evolving linguistic practices, such as translanguaging, but little attention has been given to this issue in English as a foreign-language (EFL) classes, such as in Saudi Arabia, where there is a shortage of research examining translanguaging in universities even under normal circumstances [4].

Translanguaging in this context simply refers to using the students’ L1 (Arabic) in an otherwise mostly English-medium higher education context. MacSwan [5] considered translanguaging as a modern concept in the field of bilingual education. Kamwangamalu [6] defined it as “the purposeful pedagogical alternation of languages in spoken and written” discourse (p. 2). Scholars have described it as a process that allows bilingual speakers to choose meaning-making features and freely combine them to potentialize meaning-making, cognitive engagement, creativity, and criticality [7]. In translanguaging, alternation between languages is seen as a flexible and systematic use of linguistic repertoires to gain more knowledge in a learning environment. Although code-switching and translanguaging are similar in practice, they differ in how scholars have looked at them in classroom and literacy practices; translanguaging has been received in more positive terms than code-switching as the former offers more social space for bilingual students, freeing them from keeping their languages separate for sociolinguistic reasons, which could affect their language performance [8].

Translanguaging has been examined in terms of its efficiency, practicality, and how it promotes or challenges the educational and societal goals of multilingual contexts across the world [5, 9, 10]. For instance, researchers have discussed how language teachers use their linguistic resources to enhance the learning experience [11]. They have also examined whether code-switching is more beneficial and have investigated linguistic practices in language classrooms from different perspectives to identify the best practices in a digital context. Beres [9] confirmed that some teachers face difficulty meeting learners' needs due to language issues and turn to mixing learners' first language (L1) with the second language (L2), especially with low-proficiency learners. Examining teachers' perspectives would reveal more about translanguaging and how teachers’ attitudes and awareness about this approach shape learning experiences. To meet the needs of multilingual societies in the 21^st^ century, “it is necessary to shift from approaching bilingualism, as two separate, rigid and static languages, to viewing them as fluid, flexible and permeable” (p 104). In this way, translanguaging is associated with the fluidity of language use in settings where teachers and students can build on their linguistic repertoire. This study adopted this view of translanguaging as a starting point to understand how and when it is used in bilingual higher education.

Many studies have shown translanguaging to improve academic success [9], increase student autonomy [12], and show the value of linguistic diversity [5]. However, as this approach is relatively new, there is a lack of research on the most effective ways to implement it in the classroom [13]. Therefore, this study tries to bridge this gap by exploring the use of translanguaging by EFL instructors in the actual classrooms. It may be noted here that previous studies have examined translanguaging in different settings but with more focus on K-12, where the community and education system embrace multilingualism. Although researchers are paying more attention to higher education [14], there is still less research available on translanguaging in higher education and among English teachers at universities where the community is monolingual and the spaces for negotiating bilingualism are limited to language classrooms, such as Saudi Arabia and other Arab countries. Thus, the present study seeks to address this gap by examining teachers’ attitudes toward translanguaging in higher education in a monolingual community and a non-internationalized education system in Saudi Arabia.

Several studies have addressed translanguaging in English language classroom, but there is not enough research on teachers’ practices in higher education [15, 16], with even fewer in digital contexts [17]. Therefore, this study chose to investigate translanguaging among bilingual teachers in online university EFL classes. Guided by the Language Policy Model [10, 18, 19] view of translanguaging as a pedagogy, the study sought to answer the following research questions:

1. Do bilingual EFL teachers use translanguaging? Why and under which circumstances?
2. What factors do bilingual EFL teachers consider when deciding to use translanguaging?
3. What are bilingual EFL teachers' perspectives on translanguaging in online classes?
4. What advantages do bilingual EFL teachers see in translanguaging?

## 2 Literature review

### 2.1 Translanguaging and pedagogy

New theories in bilingualism have contributed to the emergence of *translanguaging*, questioning the previous findings of language boundaries and restrictions [20, 21, 22]. The term translanguaging was coined in the 1980s [23,24] and has seen development more recently in response to changes occurring in different educational settings [25]. García and Wei [7] stated that *translanguaging* is effective in not only simplifying language exchange among people but also establishing an understanding of language identify that is dominant in a state. Furthermore, García [8] described *translanguaging* as a pedagogical approach with the potential to liberate people's voices and encourage them to think outside of conventional academic contexts. This could help learners gain new perspectives and momentum for understanding social structures and relationships. Wei [26] came to similar conclusions stating that translanguaging shapes a communal place which enables multilingual users to present their historical background, and experiences. Other studies [27] have referred to translanguaging as a natural practice by bilinguals that has the potential to be an effective tool for teachers and students. Allard [27] conducted an autoethnographic study that employed two ESL teachers at a high school where English and Spanish were used. The study found that translanguaging made communication between teachers and students easier, helped students with limited English fluency participate in their L1, and validated their previous knowledge and language skills. The latter finding is supported by Hillman *et al* [4], who reported that translanguaging practices were effective at making a connection between the languages used at home and at school. They added that translanguaging had a positive impact on classroom management and students' cognitive and linguistic development. It also validated and valued students' linguistic identities.

In a similar vein, several studies [21, 22] have explained translanguaging as a process in which a speaker uses their linguistic repertoire to perform various tasks in two or more languages. These tasks, according to Creese and Blackledge [21], include conveying information, creating meaning, and presenting identities. Employing translanguaging pedagogy requires instructors to help students be aware of their linguistic abilities and learn to channel them in different situations for different purposes [16, 28].

### 2.2 Translanguaging in higher education

According to Mazak and Carroll's [14] study abroad programs, international students' social mobility, and massive increases in tuition tell a lot about recent changes in higher education. Such changes have had a particularly strong effect in situations where different cultures and languages interact. In one of the few studies on translanguaging in higher education, Mazak and Carroll [14] investigated the language policy at several universities in Puerto Rico with a focus on one undergraduate psychology class. Most of the universities had no clear policies for the language of instruction; however, a few indicated their position was “open,” allowing professors to implement their own micro-level policies. This created fertile ground for translanguaging. One interesting finding was that trying to enforce a monolingual language ideology could produce resistance from the local language/culture, which in turn could result in classrooms employing more translanguaging. Kagwesage [29] investigated strategies that students used to facilitate comprehension in a foreign language and promote learning. The study took place in a university in Rwanda where English was used as the medium of instruction. The use of translanguaging to mediate challenging content was one of the top strategies found. Although Rwandan students' L1, Kinyarwanda, was not the official medium of instruction, according to García [8] its mediating role and potential to facilitate was given more attention by permitting responsible code-switching and translanguaging. Few studies have focused on translanguaging in higher education; instead, it is typically discussed in studies about language policy. Adamson *et al* [30] investigated a Self-Access Learning Center in a Japanese university from a qualitative perspective by interviewing students, teachers, staff, and management. The center changed its “English only” policy to use translanguaging and showed more language learning diversity just two years after its opening.

### 2.3 Translanguaging in the arab world

Research on translanguaging is scarce in the Arab world, particularly in higher education. Dillon [31] looked at experiences of co-teaching within a new bilingual (Arabic/English) model in public kindergartens in the United Arab Emirates and noted that few studies had looked at Arabic-English biliteracy [32, 33]. Dillon and Gallagher [31] interviewed six pairs of kindergarten co-teachers in Abu Dhabi. They found that different classroom practices, such as translanguaging and class management, were enhanced by co-teaching, while flexible translanguaging, in turn, supported co-teaching and biliteracy outcomes. Aghai [34] investigated the ideologies of English as a second language (ESL) teachers regarding their students' translingual practices and to what level they would allow adult students to use their L1 in the classroom. The researcher observed four ESL classes and conducted follow-up interviews with students and teachers. The results were in line with previous studies [35] which concluded that translanguaging occurs in ESL classes for multiple purposes, such as clarification, checking for comprehension, and feedback. Lower-level students used translanguaging to build fluency, while more advanced students used it to build accuracy. Two groups of teachers employed different strategies: A monolingual group practiced a lack of control in approaching students' translanguaging, while a multilingual group was more tolerant of L1 interference and considered students' use of their native language to be natural and a valuable resource. ESL teachers could benefit from their metalinguistic skills and translingual competence to build on students' linguistic resources [36]. However, there is a need to expand the understanding of translanguaging in higher education in Arab countries. Previous literature shows that most of these have been conducted in contexts where multilingualism is common outside the classroom, but few have focused on monolingual contexts where bilingualism is only negotiated in the classroom. The current study sought to address these gaps while aiming to highlight teachers' use of bilingualism to communicate with students, motivating them to learn ESL and helping them engage in pedagogical processes inside and outside the classroom. The study explores the advantages of translanguaging in Saudi EFL university classes and teachers’ perspectives on translanguaging in a normally monolingual context.

## 3 Methods

### 3.1 Research design

A mixed-methods design was applied in this study. Mixed methods research pertains to collection of both quantitative and qualitative data and interpreting them in context during the analysis process to answer the research questions. A 23-item five-point Likert Scale questionnaire was applied to collect the quantitative data. Video-recorded sessions and semi-structured interviews were conducted to collect the qualitative data from the participants.

### 3.2 Participants

**Figure 1:**
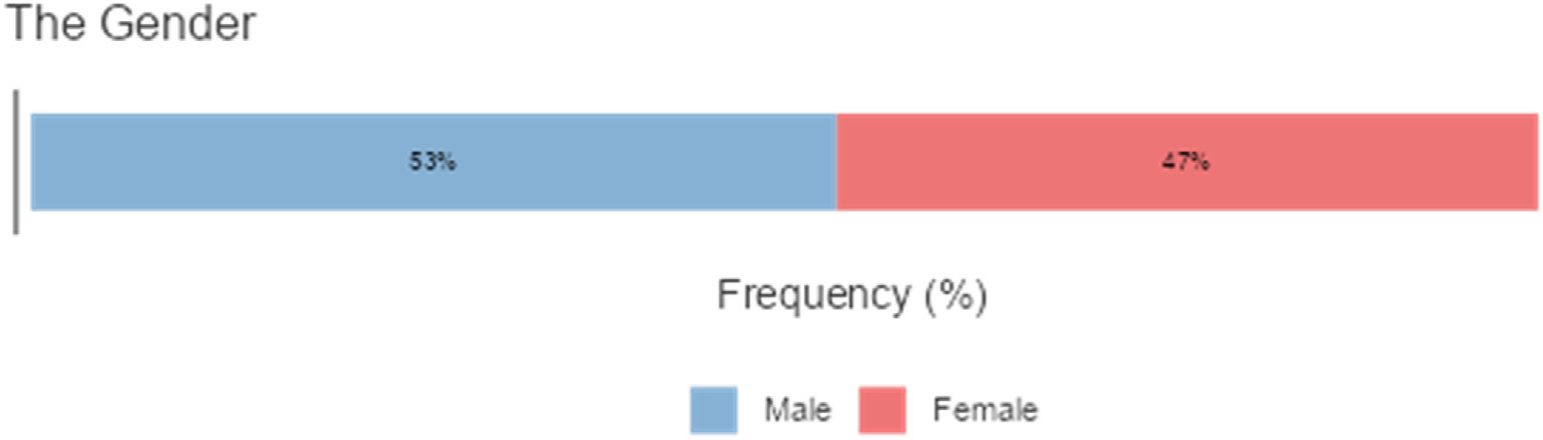
Percentages of participants according to the Gender.

**Figure 2:**
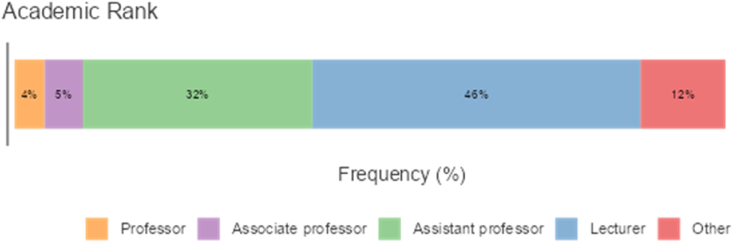
Percentages of participants according to academic rank.

**Figure 3:**
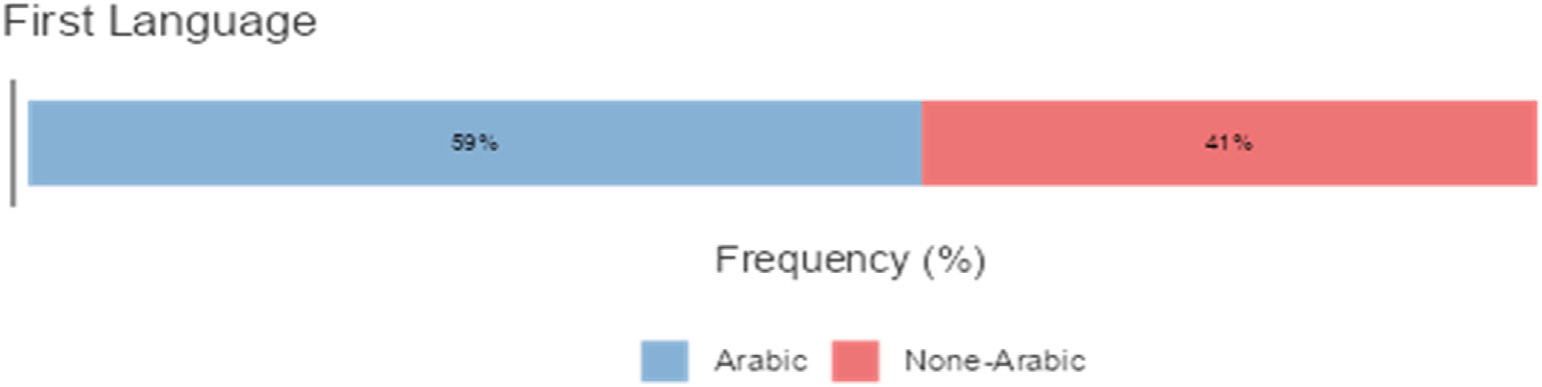
Percentages of Participants According to language they speak.

**Figure 4:**
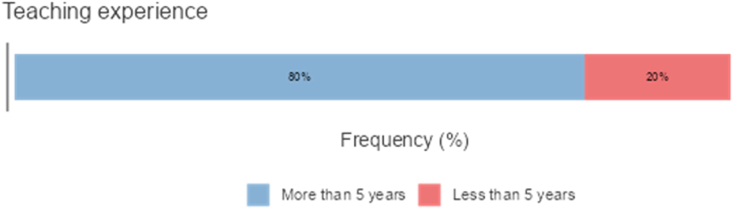
Percentages of Participants According to the teaching experience.

The study targeted a homogenous sample of 260 teachers in Saudi Arabia. Of these participants, 59.2% were native Arabic speakers, and 40.8% were none-Arabic speakers. The first group comprised only Arabs whereas the second group was from India and Pakistan, and Bangladesh. Among the second group there were a few teachers from UK and all the group of teachers were teaching at various Saudi Universities such as at the University of King Abdul Aziz at Jedda and University of Bisha. Participant demographics are presented (see Figures 1, 2, 3, and 4). The participants were selected through purposive sampling to ensure that the participants could furnish the information that fulfilled the specific needs of the study [37]. Teachers teach courses and classes related to the language skills and language components. They filled out the survey, which was sent to them by email or WhatsApp. Five bilingual instructors took part in the interviews, and 26 video-recorded classroom sessions were observed. Details about the interview participants are given (see Table 1) Research ethics were maintained throughout this research. The researcher obtained approval letters from the ethic research committee at the University of Bisha numbered (UB-16-1442). Participants were also informed about the purpose of the research, and they verbally agree that they would participate in the study and their information may be published. Such verbal consent is approved of by the committee of ethic in the university.

**Table 1:** Interview participant information.

Interviewee	Experience	Age	Academic Rank
1	20 years	45–60	Associate Professor
2	12 years	35–45	Assistant Professor
3	10 years	35–45	Assistant Professor
4	15 years	30–40	Lecturer
5	18 years	35–45	Lecturer

### 3.3 Data collection

The data were collected through a survey (see Appendix A), video-recorded sessions of online classes, and semi-structured interviews (see Appendix B). The survey was designed with variables targeting teachers’ practices, perspectives, and perceived advantages of translanguaging. The researchers employed the survey from Hillman *et al.* [37] with slight modifications. The final instrument contained 23 questions on a 5-point frequency scale (5 = always, 4 = often, 3 = sometimes, 2 = rarely, 1 = never) for the first two variables and for the rest, another five-point Likert Scale was applied (1 = strongly disagree, 2 = disagree, 3 = neutral, 4 = agree, 5 = strongly agree). Open-ended questions were included in the survey to obtain more in-depth information.

The video sessions were automatically recorded. The researchers requested some participants (teachers) to volunteer the use of their recorded session for the purpose of the research (see Table 1). They welcomed the idea and shared the links of the videos with the researchers. The interviews were conducted at the University of Bisha through a process of volunteering after a common email to the effect was sent out to the teachers in the university database. Due to COVID-19, all interviews were conducted online via Blackboard, Zoom, or Google Meet, depending on each participant's preference and convenience.

### 3.4 Data analysis

The study gathered quantitative and qualitative data. The quantitative data from the survey were coded as (5 = strongly agree/always & 1 = strongly disagree/never) using SPSS (Version 29). For the quantitative data, descriptive analysis was performed by computing the mean scores and standard deviations. The qualitative data comprising the open-ended survey questions, online video-recorded class sessions, and interviews—were thematically analyzed. The level of analysis used is the sentence. Number codes from (1 into 25) were attached with the participants in Tables 4, 6 below. The first part of the survey collected demographic information, including teaching experience, gender, academic rank, L1, and L2. Furthermore, the reliability of the questionnaire items was calculated on the second part of the survey. Cronbach's alpha scored .*77* for the 23 items indicating that their reliability and consistency were acceptable.

The second part of the survey targeted the importance and frequency of translanguaging in the classroom. The qualitative data helped the researchers determine attitudes about translanguaging and support the quantitative findings. All 26 recorded sessions were reported. The researchers first examined the use of the Arabic language (e.g., whether participants were using it for instruction or explaining vague and complex ideas in class or during office hours). The interview analysis focused on teachers’ perspectives on how the L1 could be useful in EFL learning. Thematic analysis was then applied to the data for identifying, analyzing, and reporting themes, i.e., “something important about the data in relation to the research question [that] represents some level of patterned response or meaning” [37]. Themes were identified in initial and focused coding [38]. During *initial coding*, i.e., dividing the data into smaller units and comparing them, the researchers studied “fragments of data—words, lines, segments, and incidents—closely for their analytic import [38]. When engaging in focused coding, the researchers selected what appeared to be the most useful initial codes and tested them against extensive data. Throughout this process, as suggested by Charmaz [38], the researchers compared data with data and then data with codes.

## 4 Results

This section discusses (a) the frequency and functions of translanguaging by the bilingual EFL teachers, (b) teachers’ perspectives, and (c) advantages of translanguaging in a monolingual (Arabic) context.

### 4.1 Frequency and functions of translanguaging

Table 2 presents the frequency of translanguaging in online classrooms. The average mean score of the participants’ allowance for using Arabic during the EFL class rated (*M* = 3.50, *SD* = 0.98). This average score (3.50) on a five-point scale is consider medium. The highest mean score was reported for, “I allow my students to answer questions orally in Arabic” (M = .4.13, SD = 0.98). On the other hand, the lowest means were for using Arabic to build a rapport with students in situations like small talk, where the frequency was high (*M* = 3.26,*SD* = 1.01). Overall, Arabic was common during different classroom practices, such as explaining concepts, giving directions, online teaching, answering questions, and discussions. Teachers tended to use translanguaging online and in face-to-face classes (see Table 2).

**Table 2:** Use of translanguaging in online classrooms.

Question	*M*	*SD*
I explain academic concepts orally in Arabic.	3.35	0.899
I use Arabic for building rapport with students (e.g., small talk).	3.26	1.001
I use Arabic for classroom management purposes (e.g., giving instructions).	3.28	1.098
I use Arabic in face-to-face teaching.	3.47	0.948
I use Arabic in online classroom teaching.	3.39	0.938
I allow my students to answer questions orally in Arabic.	4.13	0.983
I allow students to answer questions in writing in Arabic.	3.68	0.998
I allow my students to use Arabic to discuss something with a classmate.	3.39	0.934
Average	3.508	0.980

The survey, interview, and session recordings showed that bilingual teachers used translanguaging for limited purposes in certain situations. As shown in Table 3, when the participants were asked about their use of Arabic in class, 24 out of 54 (44%) reported using it to explain concepts that were difficult or important. Some (18%) mentioned using Arabic mainly in beginner and freshmen classes. More common themes included the use of Arabic in Translation and Comparative Studies (14%) and to explain instructions for exams and quizzes (12%). The smallest group (9%) reported not using Arabic in the classroom and not recommending using it at all. On many occasions, teachers switched to the students’ L1, finding it useful for explaining difficult terms, managing class, and class discussion.

**Table 3:** Arabic in class.

When do instructors use Arabic inside the classroom?	Sample Quote	*N*
To explain difficult and/or important concepts	“For explaining difficult concepts or new words.”	24
L1 is used in beginner classes	“I would consider using Arabic inside the classroom if I have low-achieving students whose level is beginner or below.”	10
L1 is used in Translation and Comparative Studies	“In comparative studies, I try to explain the Arabic section in Standard Arabic after explaining it in English.”	8
To explain assessment guidelines	“I use Arabic only when giving exam instructions.”	7
L2 should not be used at all inside the classroom	“Arabic shouldn't be used at all inside classrooms.”	5

Teachers reported using translanguaging more during office hours than in class. This was supported by the rest of the data, which showed Arabic was less often used in class (0–27%) than in office hours (0–52%). Table 5 presents statistics for Arabic use during office hours with an average of (M = 3.01, SD = 1.241). Although the means were close, the highest was for allowing students to answer questions using Arabic (*M* = 3.08, *SD* = 1.28) compared to building rapport (*M* = 2.95, *SD* = 1.24). Teachers thus showed more flexibility with translanguaging during office hours (see Table 4).

**Table 4:** Translanguaging during office hours.

Question	*M*	*SD*
I often explain academic concepts orally in Arabic during outside virtual classrooms.	3.02	1.189
I often use Arabic for building rapport with students (e.g., jokes, small talk) during virtual classroom.	2.95	1.248
I allow my students to answer questions orally in Arabic in virtual classrooms in office hours.	3.08	1.286
Average	3.01	1.241

In response to the question on when they used Arabic during office hours, almost half the participants (47%) reported that they tended to use it with students at the beginning level because those students lacked the skills to communicate effectively in English (see Table 5). Another theme, reported by 37%, was the use of Arabic in social settings or to discuss personal issues unrelated to class. A small percentage (10%) stated they only used the L1 when there was something important to talk about outside the classroom. Only two (7%) reported using the L1 when students came to their office hours. There was a tendency to use the L1 to explain difficult concepts or communicate important information.

**Table 5:** Arabic outside of class.

When do instructors use Arabic outside the classroom?	Sample Quote	*N*
L1 is used in social settings or to discuss personal matters	“When talking about personal things or when talking about problems preventing students' educational progress, such as problems outside the university.”	11
L1 is used with low-proficient students	“If they are not able to communicate in English, I try to use a little Arabic that I know.”	14
L2 is used during office hours	“Office hours.”	2
To explain something important	“Almost all the points I discuss with the students outside the class are important and they have to understand it well.”	3

**Figure 5:**
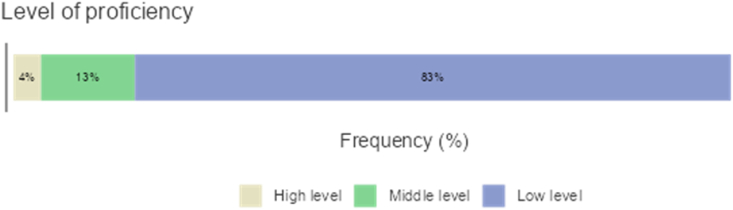
English Proficiency at which Translanguaging is used.

Figure 5 shows that bilingual teachers used translanguaging more in classrooms with less proficient learners. The teachers believed such use of Arabic was more productive in the early stages of L2 learning to motivate students, engage them in activities and discussions, and facilitate deeper understanding of complex concepts. However, exclusive use of the L2 could lead to gaps in understanding of the subject matter [39, 40, 41]. On the other hand, some bilingual teachers tended to avoid switching to the L1, believing it could hinder understanding of the L2, lead to a dependency on the L1, and reduce L2 exposure [42, 43].

## 5 Teachers’ perspectives

Table 6 summarizes teachers' perspectives on translanguaging in EFL classes. The teachers’ ideologies were consistent with their practices, as they expressed moderate general agreement with an average of (M = 3.21, SD = 1.10) that Arabic could be helpful to explain concepts, build a rapport with students, manage the classroom, facilitate online and face-to-face teaching, and answer questions. There was low agreement on using translanguaging during writing classes, but this does not necessarily contradict that the teachers believed in using translanguaging. The teachers believed the L1 strengthened the learning process through student discussion.

**Table 6:** Teachers’ perspectives on translanguaging.

Item	*M*	*SD*
I think Arabic can be used in English language learning to explain academic concepts.	3.48	1.03
I think Arabic can be used to build rapport with students (e.g., small talk).	3.56	0.986
I think Arabic can be used for class management.	3.5	0.996
I believe that Arabic can be used more in face-to-face teaching.	3.19	1.125
I believe that Arabic can be used more in online classrooms.	3.23	1.091
I believe that Arabic can be used to answer questions orally.	2.98	1.24
I believe that Arabic can be used to answer questions in writing.	2.43	1.27
I believe that students can use Arabic to discuss things with their classmates.	3.37	1.063
Average	3.21	1.10

### 5.1 Advantages of translanguaging

Table 7 presents the possible advantages of translanguaging in L2 classes. Again, there was moderate agreement with an average mean score of (M = 3.36, SD = 1.04) that translanguaging enhanced learning. According to participants, it increased student engagement, motivation, and comprehension and decreased pressure on them.

**Table 7:** Advantages of translanguaging.

Item	*M*	*SD*
Using the L1 in class helps increase the students' engagement.	3.41	1.056
Using Arabic enhances students' motivation for learning EFL.	3.24	1.069
Using Arabic in class increases the students' understanding of the subject matter.	3.43	1.017
Using Arabic makes the students more secure and comfortable to learn EFL.	3.37	1.052
Average	3.36	1.04

Based on the open-ended survey questions (see Table 8), compares teachers' opinions about the reported advantages and disadvantages of using students’L1 in the classroom with illustrative quotations. The advantages included the following:

⁃ Helping students develop confidence and reduce anxiety (23%).
⁃ Helping less-proficient students keep up with more-proficient students (23%).
⁃ Enhancing L2 learning (27%).

**Table 8:** Advantages and Disadvantages of Translanguaging.

Common Themes with Quotes	*N*	Common Themes with Quotes	*N*
**Helping students develop confidence and reduce anxiety.** “Using first language in the classroom counted as a secure option that reduces foreign language anxiety since anxious learners can rely on it when things get hard in the class.”	5	**Hindering students' L2 fluency.** “It mainly hinders the students' ability to improve their skills in the L2 where they always remain waiting for the support of the first language. They will always remain dependent when it comes to activating and using the L2. They will hardly be able to think in the L2 and use it naturally.”	15
**Helping less-proficient students keep up with more-proficient students**. “Using Arabic saves class time and involves low-achieving students in the learning process.”	5	**Making students dependent on the L1.** “It increases dependence on the medium of expression and lessens the willingness to take risks, which is a necessary requirement for learning a new language.”	8
**EnhancingL2 learning.** “The use of mother tongue in the class will enhance learning.”	6	**Lessening students' chances of practicing the L2.** “There is no chance for the students to practice the language.”	2
**No advantages.** “Using Arabic does not bring about any benefits.”	6		
Total	22		25

Around a quarter of participants (27%), however, did not believe the students’ L1 offered any advantages in the L2 classroom. Regarding disadvantages, the majority (60%) felt the main problem was that translanguaging could negatively affect learning outcomes and that students were less likely to develop a stronger proficiency. Around a third (32%) were concerned that using the L1 was likely to make students dependent on their L1. Two of the instructors (8%) believed that if they allowed students to use the L1, they would not have enough opportunities to practice the L2.

## 6 Discussion

The survey, interviews, and classroom observations generally showed that ESL university teachers in Saudi Arabia used translanguaging in online classrooms and during virtual office hours. Teachers exhibited the belief that translanguaging was useful in certain contexts for various academic purposes, despite some teachers, universities, and policymakers believing English should be the sole medium of instruction. The findings are in line with recent studies highlighting how translanguaging can enhance the learning process [44, 45, 46]. The first research question asked, “Do bilingual EFL teachers use translanguaging? Why and under which circumstances?“The study found that teachers actually used various translanguaging practices for classroom management, giving instructions, providing feedback, and explaining complex terms and vague concepts. Similar results have been found in other contexts in Arab countries [47, 48, 49]. Translanguaging was used during virtual office hours to explain complex concepts and terms, give feedback, and discuss social matters. These results are similar to those of Rabbidge's [50] study, highlighting the role of teachers' translanguaging in students' ability to participate, understand teachers and activities, and engage in an interesting and interactive environment.

With respect to the second research question (What factors do bilingual EFL teachers consider when deciding to use translanguaging?), teachers used translanguaging in various academic contexts for pedagogical purposes, considering the English proficiency of the students. The quantitative and qualitative analysis indicated that translanguaging was used to a great extent with students who had lower proficiency in English and that it could motivate such students to engage in classroom activities. Such advantages have been reported in many similar contexts [51, 52, 53, 54]. This would suggest that using English only might be less effective in the early stages of learning, especially for learners with lower language skills. Moreover, the mixing of the L1 and L2 changed over time according to various factors, such as teacher preference and student proficiency. Translanguaging occurred more often outside the classroom due to the less formal nature of interaction, similar to other studies [4, 55].

Concerning the third research question (What are bilingual EFL teachers’ perspectives on translanguaging in online classes?), teachers reported having positive perspectives about translanguaging and used their linguistic repertoires according to the context. Teachers found that translanguaging, though used rarely according to the data, was helpful inside and outside the classroom. They believed that using Arabic could reinforce learning by improving student participation and discussion, explaining complex concepts and terms, class management, and giving instructions for exams and activities. The findings were also similar to previous studies in that bilingual teachers tended to use the L1 (Arabic) to simplify complex terms [56] and improve learner skills [52]. Similarly, Rabbidge [52] explained how translanguaging helped engage students in classroom learning and sustainable practice in language contact situations [57]. Regarding the fourth research question (What advantages do bilingual EFL teachers see in translanguaging?), the study found some advantages of translanguaging, although the use of translanguaging was rare due to the English-only policy of the institution. For example, translanguaging could help students with low English proficiency engage in activities and discussion, clarify terms and concepts, make learning less stressful, and reduce learner anxiety, in line with previous studies [58, 59, 60].

## 7 Conclusion

This study sheds light on how EFL teachers in Saudi Arabia perceive and practice translanguaging in online classrooms and virtual office hours. The quantitative survey data and qualitative data (classroom observations, open-ended survey questions, and interviews) showed that translanguaging was used by those bilingual teachers who viewed it as useful in online and face-to-face classes. Positive functions of translanguaging included understanding complex terms, class management, and discussion. Furthermore, teachers held positive opinions of it in online classrooms in a monolingual context, as the L1 enhanced the learning process, a finding which is in line with other studies [52, 56]. Overall, translanguaging [61] was more common during office hours. The teachers' use of translanguaging to facilitate knowledge construction and classroom management is in line with Ferguson's [61] categorization of using the L1 in the classroom. However, the present study showed translanguaging could include office hours and online classes. Ferguson's [61] third category, interpersonal relations, was not evident in this study, which might be attributed to the nature of online classroom communication. The amount of translanguaging varied depending on learner proficiency, being more common with lower levels of proficiency, as most participants believed translanguaging increased student engagement and motivation, especially at lower levels.

Overall, this study has revealed several pedagogical advantages and implications of translanguaging over a traditional monolingual approach. Translanguaging creates a good and dynamic learning atmosphere where students' L1 can be exploited to improve students' understanding of the course content and creates a positive learning environment. The findings could thus be used to inform education policy makers, curriculum designers to recognize the importance of translanguaging practices in higher education. Furthermore, the study could act as a starting point for exploring translanguaging in an Arabic monolingual context where the L2 is seldom spoken outside the classroom. Future studies could build on this by comparing how teachers and students use and perceive translanguaging. One limitation of the study is that some participants in the survey were non-Arabic speaking teachers, and this could be taken for further studies to address and examine the implementation of translanguaging among non-Arabic native teachers in multilingual contexts. Furthermore, future research studies can explore students' perceptions about teachers' use of translanguaging and compare it with the teachers’ perceptions about to provide more in-depth results about the nature and functions of translanguaging pedagogical implications and practices.

## Declarations

### Author contribution statement

Muhammad Alasmari: Conceived and designed the experiments; Performed the experiments; Analyzed and interpreted the data; Wrote the paper.

Fawaz Qasem: Conceived and designed the experiments; Performed the experiments; Analyzed and interpreted the data; Wrote the paper.

Rashad Ahmed: Performed the experiments; Analyzed and interpreted the data; Wrote the paper.

Muhammad Alrayes: Conceived and designed the experiments; Performed the experiments; Wrote the paper.

### Funding statement

The authors extend their appreciation to the Deputyship for Research & Innovation, Ministry of Education in Saudi Arabia for funding this research work through the project number (UB-16-1442).

### Data availability statement

Data included in article/supp. material/referenced in article.

### Declaration of interest's statement

The authors declare no conflict of interest.

### Additional information

No additional information is available for this paper.

## Appendices Appendix A: survey

**The following questions ask about the teacher's use of Arabic during classes**.

1. How often do you explain academic concepts orally in Arabic?
2. How often do you use Arabic for building rapport with students (e.g., small talk)?
3. How often do you use Arabic for classroom management purposes (e.g., giving directions)?
4. How often do you use Arabic in face-to-face teaching?
5. How often do you use Arabic in online classroom teaching?
6. How often do you allow your students to answer questions orally in Arabic?
7. How often do you allow students to answer questions in writing in Arabic?
8. How often do you allow your students to use Arabic to discuss with a classmate?
9. Overall, what percentage of time do you use Arabic in your class? Kindly write the percentage ______ %
10. The students' level of proficiency when Arabic is used
  (a) High level of English proficiency
  (b) Middle level of English proficiency
  (c) Low level of English proficiency
11. Kindly write briefly some points when and for what purpose you consider using Arabic inside classrooms. (Open-ended question)

**The following questions target the use of Arabic during interaction outside the classroom**.

12. How often do you explain academic concepts orally in Arabic during virtual office hours?
13. How often do you use Arabic for building rapport with students (e.g., jokes, small talk) during virtual office hours?
14. How often do you allow your students to answer questions orally in Arabic during virtual office hours?
15. Overall, what percentage of time do you use Arabic with students during your online interaction with students outside the classroom? ______ %
16. Kindly write briefly some points when and for what purpose you use Arabic outside the classroom during interaction with learners. (Open-ended question)

**The following questions ask about the teachers’ perspectives on the use of Arabic**.

17. I think Arabic can be used in English language learning to explain academic concepts.
18. I think Arabic can be used to build rapport with students (e.g., small talk).
19. I think Arabic can be used for class management.
20. I believe that Arabic can be used more in face-to-face teaching.
21. I believe that Arabic can be used more in online classrooms.
22. I believe that Arabic can be used to answer questions orally.
23. I believe that Arabic can be used to answer questions in writing.
24. I believe that students can use Arabic to discuss with their classmates.
25. Kindly write some points on your ideologies on the use of Arabic inside the classroom. (Open-ended question)

**The following questions ask about the advantages of using Arabic in EFL classes**.

26. Using the L1 in class helps increase students' engagement.
27. Using Arabic enhances students' motivation for learning EFL.
28. Using Arabic in class increases students' understanding of the subject matter
29. Using Arabic makes the students more secure and comfortable to learn EFL
30. Kindly if you have more important points on the advantages of using Arabic in classrooms, please add them. (Open-ended question)
31. Kindly write the shortcomings and the disadvantages of using the L1 while teaching in classrooms. (Open-ended question)

## Appendix B Simi structural interview protocol questions

1. In general, how do you feel about using Arabic in the online classes?
2. How do you feel also about using Arabic in the online classes outside classrooms during your interaction with learners?
3. This is from a day I observed a teaching session (show video segments). Can you explain why you chose to use Arabic in those moments?
4. Can you give some examples of when you have used Arabic in the class previously and why?
5. What other factors do you consider when choosing to use Arabic in your classroom?
6. Do you think using Arabic can be beneficial as a pedagogical technique?
7. Do you think that using Arabic technique/strategies should be included in the curriculum in designed tasks/activities?
8. How do you feel about students using Arabic in your class? When would you allow it and when would you not?
9. How often do you use Arabic with students outside classrooms during interaction with learners?
10. What factors do you consider when you use Arabic outside classrooms during interaction with learners?
11. Can you give some examples of when you have used Arabic outside classrooms during interaction with learners?
12. Why do you feel that there is a need to use Arabic in the classrooms?
13. When do you feel the need to use Arabic?
14. Why do you feel that there is no need to use Arabic in classrooms?
15. Why do you think teachers do not use Arabic in classrooms?
16. In which classrooms, is Arabic used more, face-to-face or virtual/online classrooms?
17. Why do you think teachers may use Arabic more in online classrooms?
18. Why do you think teachers may use Arabic more in face-to-face classrooms?
